# Biomarkers for Exposure as a Tool for Efficacy Testing of a Mycotoxin Detoxifier in Broiler Chickens and Pigs

**DOI:** 10.3390/toxins11040187

**Published:** 2019-03-28

**Authors:** Marianne Lauwers, Siska Croubels, Ben Letor, Christos Gougoulias, Mathias Devreese

**Affiliations:** 1 Department of Pharmacology, Toxicology and Biochemistry, Faculty of Veterinary Medicine, Ghent University, 9820 Merelbeke, Belgium; marianne.lauwers@Ugent.be (M.L.); Mathias.Devreese@ugent.be (M.D.); 2 Innovad, Postbaan 69, 2910 Essen, Belgium; B.Letor@innovad-global.com (B.L.); C.Gougoulias@innovad-global.com (C.G.)

**Keywords:** biomarkers, exposure, efficacy, mycotoxin detoxifier, pig, broiler chicken

## Abstract

Applying post-harvest control measures such as adding mycotoxin detoxifying agents is a frequently-used mitigation strategy for mycotoxins. EFSA states that the efficacy of these detoxifiers needs to be tested using specific biomarkers for exposure. However, the proposed biomarkers for exposure are not further optimized for specific target species. Hence, the goal of this study was (a) to evaluate the most suitable biomarkers for deoxynivalenol (DON) and zearalenone (ZEN) in porcine plasma, urine and feces; and DON, aflatoxin B1 (AFB1) and ochratoxin A (OTA) in plasma and excreta of broiler chickens and (b) to determine the efficacy of a candidate detoxifier, as a proof-of-concept study. Therefore, a mixture of mycotoxins was administered as a single oral bolus with or without detoxifying agent. In accordance with literature AFB1, OTA, and DON-sulphate (DON-S) proved optimal biomarkers in broilers plasma and excreta whereas, in pigs DON-glucuronide (DON-GlcA) and ZEN-glucuronide (ZEN-GlcA) proved the optimal biomarkers in plasma, DON and ZEN-GlcA in urine and, ZEN in feces. A statistically significant reduction was seen between control and treatment group for both AFB1 and DON in broiler plasma, under administration of the mycotoxin blend and detoxifier dose studied suggesting thus, beneficial bioactivity.

## 1. Introduction

Broiler chickens and pigs are highly exposed to mycotoxins due to their cereal based diet. These toxins are mainly produced by *Aspergillus*, *Fusarium*, and *Penicillium* fungal species. [1]. Aflatoxin B1 (AFB1) is the most important aflatoxin with regards to potency and occurrence. Poultry are highly sensitive to the hepatotoxic and hepatocarcinogenic effects of AFB1. At lower doses, reductions in growth rate, hatchability, feed efficiency, and immunity occur, which result in economic losses [2]. Ochratoxin A (OTA) has toxic effects on numerous animal species and especially targets the kidney. Next to nephrotoxicity, OTA can also induce immunosuppression, teratogenicity and mutagenicity in poultry [3]. Ochratoxicosis has been detected at contamination levels of 2 mg/kg in feed with symptoms ranging from body weight (BW) loss, decreased egg production, increased water intake to diarrhea [4]. Deoxynivalenol (DON) is one of the major *Fusarium* mycotoxins contaminating 80% of feed samples worldwide [5]. It impairs especially the health of monogastric animals. The presence of DON in poultry feed has been found to reduce feed consumption and BW and can cause lesions in the gastro-intestinal tract [6]. In pigs, animals that are especially susceptible to the toxic effects of *Fusarium* mycotoxins, reduction in feed intake and lower BW gain are typically seen. DON is also associated with alterations in the intestinal, immune, endocrine, and nervous system. Another *Fusarium* mycotoxin commonly present in pig feed is zearalenone (ZEN). ZEN possesses an estrogen-like structure and interferes with the reproductive system of the animals. In pigs, hyperestrogenism, fertility and reproductive disorders are commonly observed [7]. 

Despite several prevention strategies on the field and adequate grain storage and transport, the presence of mycotoxins cannot be avoided. Therefore, different post-harvest control measures have been developed to protect poultry and other livestock species from the harmful effects of these toxins. One such measure is adding mycotoxin detoxifying agents to the feed. These feed additives, i.e., mycotoxin binders and mycotoxin modifiers, respectively adsorb or biotransform mycotoxins in the gastro-intestinal tract. Different types of non-nutritious materials to adsorb the mycotoxins are frequently used, such as bentonite clay and aluminum silicate [8]. The efficacy and safety of these feed additives requires investigation. Literature reports usually describe the efficacy of these materials based on non-specific parameters such as organ weight, feed intake and growth performance [3,9,10,11,12,13,14,15,16]. However, the European Food Safety Authority (EFSA) states that although such non-specific parameters are useful, only specific biomarkers for exposure can prove the efficacy of such feed additives. These specific parameters are the native mycotoxins and their metabolites in feces, urine, plasma/serum/blood, tissues, and animal products (eggs and milk) [17]. To detect such a broad range of mycotoxins, a combination of liquid chromatography-tandem mass spectrometry (LC-MS/MS) and LC-high resolution mass spectrometry (LC-HRMS) is ideally used [18]. This enables the determination not only of mycotoxins for which analytical standards are commercially available, but also of other phase I and II metabolites and interaction products for which commercial standards are lacking. 

Aflatoxin B1 undergoes a wide range of biotransformation reactions in several animal species. The phase I reactions mainly include O-de-alkylation, keto-reduction, hydroxylation, and an important epoxidation. The following metabolites are formed: aflatoxin P1 (AFP1) after de-alkylation, aflatoxicol (AFL) after keto-reduction; and aflatoxin M1 (AFM1), aflatoxin Q1 (AFQ1), as well as aflatoxin B2a (AFB2a) after hydroxylation. Aflatoxin-8,9-epoxide (AFBO), the epoxidation product, is the most reactive species and binds either to DNA (generating guanine adducts) or proteins. AFB1-8,9-epoxide can also be hydrolyzed in vitro to the dihydrodiol-form. Next to these phase I biotransformation reactions, the phase II conjugation with glutathione is an important detoxification pathway [19,20,21]. 

The biotransformation of OTA is minimal. In broiler chickens, in vitro data showed that after incubation of OTA with liver microsomes a large amount of OTA remained unchanged. Nonetheless, the metabolites 4*R*-OH-OTA, 4*S*-OH-OTA, 5-OH-OTA, 7-OH-OTA, 9-OH-OTA, and dechlorinated OTA (OTB) were detected, albeit in small amounts. In broiler chickens, 7-OH-OTA appears to be the most important hydroxylated form. Similar results were seen in chicken excreta [21,22,23]. Conjugation of OTA with glucuronic acid, sulphate and glutathione are also described in literature. However, these conjugates have not yet been identified in vivo [21,24]. 

In contrast to OTA, DON undergoes different degradation and conjugation biotransformation pathways. De-epoxy-deoxynivalenol (DOM-1) is formed after microbial de-epoxidation and is especially important in ruminants, but is also found in pigs and broiler chickens [25]. In addition to the formation of DOM-1 by the microbiota of the animal, DON can also be conjugated by the animal with glucuronic acid, sulphate, or sulfonate. Remarkably, depending on the animal species, different pathways are preferred. In humans, pigs, and ruminants, glucuronidation is the major phase II metabolic pathway [26]. The most prevalent form in humans is DON-15-GlcA. This is in contrast to other animal species where DON-3-GlcA is more prevalent. In poultry, sulphation to DON-3α-sulphate (DON-S) dominates [21,25,27]. 

In vivo analysis showed that ZEN is reduced to α-zearalenol (AZEL) and β-zearalenol (BZEL) in the liver and gastro-intestinal tract of animals by 3α- and 3β- hydroxysteroid dehydrogenases, leading to an increased and reduced estrogenic activity, respectively. Again, interspecies differences are observed, since in pigs AZEL is primarily formed whereas in broiler chickens this is BZEL. These differences in biotransformation can be correlated with the sensitivity of the species to ZEN [28]. Another possibility is the reduction to ZAN, which is the precursor for the further reduction to α-zearalanol (AZAL) and β-zearalanol (BZAL). Next to these phase I transformations, also the formation of phase II glucuronide conjugates of ZEN and the phase I metabolites occurs. In swine, three main forms can be found, i.e., ZEN-14-GlcA, ZEN-16-GlcA, and ZEN-14,16-diGlcA [29]. Our group recently reported that after bolus administration of ZEN, AZEL, and ZEN-14-glucoside, only ZEN-14-GlcA is formed in pigs [30]. 

The chosen biomarker for exposure should be specific for each mycotoxin and target species, closely related to exposure and easy to detect with sensitive analytical methods validated for the matrix used [17]. EFSA has proposed different biomarkers for exposure to AFB1, DON, ZEN, OTA, and fumonisins (FBs) as shown in Table 1. However, these biomarkers are not always the most optimal for each mycotoxin. For example, EFSA indicates that DON in blood serum is a relevant biomarker for exposure, while in broiler chickens and pigs DON undergoes an extensive phase II biotransformation to DON-S and DON-glucuronide (DON-GlcA), respectively, indicating that these metabolites might be better biomarkers for exposure than DON itself [31]. Moreover, the suggested biomarkers are general and not optimized for each target species separately, although remarkable species differences are seen after administration of ZEN to broiler chickens and turkeys [32]. Turkeys show a more extensive biotransformation of ZEN to AZEL compared to broiler chickens. Differences in sensitivity for DON were also hypothesized between poultry species. Both broiler chickens and turkeys show a comparable toxicokinetic behavior, but the relative ratio DON-S/DON is different, which might explain the difference in sensitivity [27]. 

A limited number of studies have used monitoring of biomarkers for exposure to determine the efficacy of mycotoxin binders [33,34,35,36,37]. In broiler chickens, Devreese et al. [33] and Osselaere et al. [34] selected DON, and Dänicke et al. [37] selected ZEN and AZEL as biomarkers to evaluate the efficacy. Di Gregorio et al. [35] tested AFB1-lysine as biomarker for efficacy in pigs. The above-mentioned studies only investigated the influence of a binder on one mycotoxin in one biological matrix (plasma [33,34,35]). Hence, co-contamination was not taken into account. Nevertheless, worldwide surveys demonstrate that 38% of the feed is contaminated with more than one mycotoxin [5]. This co-contamination can lead to additive or synergistic effects of the different mycotoxins. Therefore, it is more relevant to assess the efficacy against a mixture of mycotoxins instead of a single mycotoxin when analyzing the efficacy of a detoxification strategy. Gambacorta et al. [38] tested the efficacy of 4 agricultural byproducts and 2 commercial binders against multiple mycotoxins (FB1, DON, ZEN, OTA, and AFB1). However, this study only analyzed the mycotoxins in one biological matrix. Furthermore, the studies of Gambacorta et al. [38], Devreese et al. [33], Osselaere et al. [34] and Dänicke et al. [37] did not include phase II metabolites as possible biomarkers in their analysis. 

Hence, the aim of this study was to determine the most suited biomarker (parent mycotoxin or phase I or II metabolites) for efficacy testing of a candidate mycotoxin detoxifier agent against a blend of mycotoxins in different biological matrices of both pigs (plasma, urine, feces) and broiler chickens (plasma and excreta). Three mycotoxins (AFB1, DON, OTA) were tested in broiler chickens and two (DON, ZEN) in pigs. 

## 2. Results

### 2.1. Biomarkers for Exposure

An overview of the metabolites and conjugation/reaction products that were detected is shown in Table 2, with the most appropriate biomarker showing the highest instrument response indicated in bold. The limits of quantification (LOQ) of DON and DOM1 in pig feces were 5 ng/g. The LOQ for DON-GlcA was set at a peak area of 100/250 mg feces. The LOQ of the other mycotoxins in all matrices can be found in supplementary Tables S2–S6. 

### 2.2. Broiler Chicken Trial

**Figure 1:**
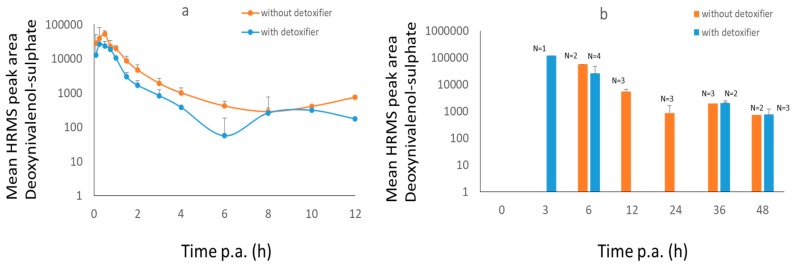
Mean high resolution mass spectrometry (HRMS) peak area-time curves (+SD) of deoxynivalenol-sulphate (DON-S) in plasma (**a**) and dried excreta (**b**) of broiler chickens after oral administration of a bolus of DON (0.5 mg/kg BW), OTA (0.25 mg/kg BW), and AFB1 (2 mg/kg BW), either with (treatment group, *n* = 8, blue curve) or without detoxifier (control group, *n* = 8, orange curve). For broiler chicken excreta it was not possible to obtain all the samples at each time point, n is shown as N above the graph bars. Samples were taken at each time point. Lacking bars means that no mycotoxins were present above the limit of detection.

After administration of DON to broiler chickens, no DON in plasma could be detected, only DON-S was found (Figure 1). The maximum concentration of DON-S was reached in plasma at 30 min post administration (p.a.) and in dried excreta after 3–6 h. A significant difference (*p* = 0.03) in area under the curve (AUC) between control and treatment group was observed in broiler plasma. This difference was not confirmed in dried excreta due to the low number of samples. This was caused by intermittent sampling of excreta, it would have been more useful to collect continuously. However, this was not possible with the present facilities. 

**Figure 2:**
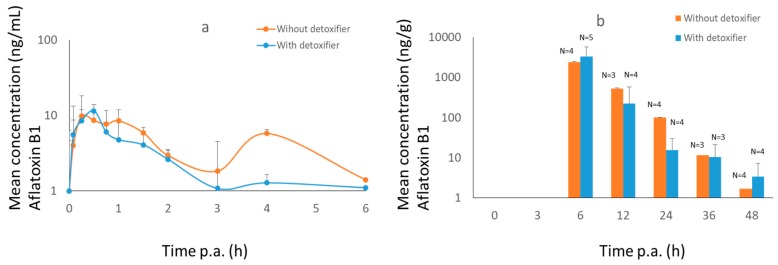
Mean concentration–time curves (+SD) of AFB1 in plasma (**a**) and dried excreta (**b**) of broiler chickens after oral administration of a bolus of DON (0.5 mg/kg BW), OTA (0.25 mg/kg BW) and AFB1 (2 mg/kg BW), either with (treatment group, *n* = 8, blue curve) or without detoxifier (control group, *n* = 8, orange curve). Samples were taken at each time point. Lacking bars means that no mycotoxins were present above the limit of detection.

**Figure 3:**
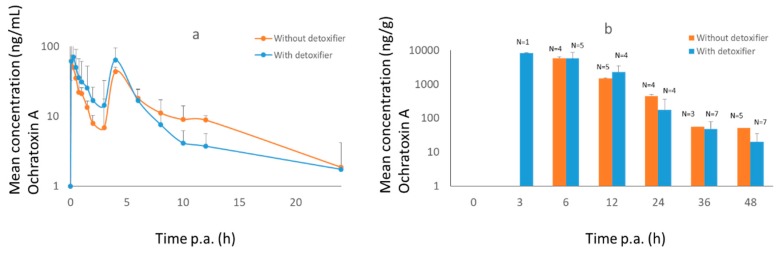
Mean concentration–time curves (+SD) of OTA in plasma (**a**) and dried excreta (**b**) of broiler chickens after oral administration of a bolus of DON (0.5 mg/kg BW), OTA (0.25 mg/kg BW) and AFB1 (2 mg/kg BW), either with (treatment group, *n* = 8, blue curve) or without detoxifier (control group, *n* = 8, orange curve). Samples were taken at each time point. Lacking bars means that no mycotoxins were present above the limit of detection.

AFB1 and OTA were both detected in plasma and dried excreta after administering AFB1 and OTA to broiler chickens (Figure 2 and Figure 3). 

The maximum concentration of AFB1 in plasma was achieved after 30 min with a second peak after 4 h, at the moment of feeding. The maximum concentration in dried excreta was found after 6 h. A significant difference in AUC between control and treatment group could be seen in plasma with a *p*-value of 0.03. In dried excreta the difference was not significant (*p* = 0.07). 

The maximum concentration of OTA was already achieved after 15 min and a second peak could be observed after 4 h. No difference could be seen between treatment and control in plasma, nor in dried excreta (*p* = 0.9 and 0.2, resp.). In Table 3 an overview is given of the toxicokinetic parameters. 

### 2.3. Pig Trial

**Figure 4:**
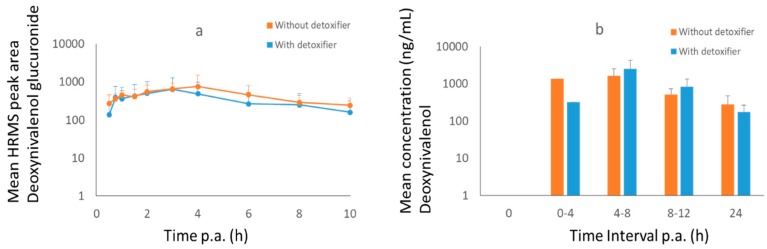
Mean high resolution mass spectrometry (HRMS) peak area–time curve (+SD) of DON-GlcA in plasma (**a**) and mean concentration-time curve (+SD) of DON in urine (**b**) of pigs after oral administration of a bolus of DON (36 µg/kg BW) and ZEN (3 mg/kg BW), either with (treatment group, *n* = 8, blue curve) or without detoxifier (control group, *n* = 8, orange curve). Samples were taken at each time point. Lacking bars means that no mycotoxins were present above the limit of detection.

After administration of DON to pigs, DON-GlcA was selected as biomarker in plasma and DON as marker in urine (Figure 4). The maximum concentration was achieved after 4 h for DON-GlcA in plasma and between 4–8 h for DON in urine. No difference could be seen between the treatment group (with mycotoxin detoxifier) and the control group (*p* = 0.9). 

**Figure 5:**
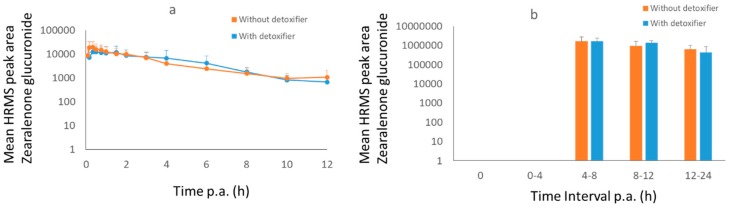
Mean high resolution mass spectrometry (HRMS) peak area–time curves (+SD) of ZEN-GlcA in plasma (**a**) and urine (**b**) of pigs after oral administration of a bolus of DON (36 µg/kg BW) and ZEN (3 mg/kg BW), either with (treatment group, *n* = 8, blue curve) or without detoxifier (control group, *n* = 8, orange curve). Samples were taken at each time point. Lacking bars means that no mycotoxins were present above the limit of detection.

**Figure 6:**
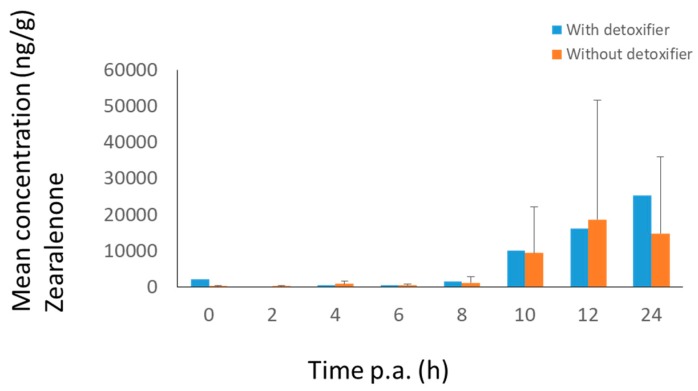
Mean concentration–time curves (+SD) of ZEN in dried feces of pigs after oral administration of a bolus of DON (36 µg/kg BW) and ZEN (3 mg/kg BW), either with (treatment group, *n* = 8, blue curve) or without detoxifier (control group, *n* = 8, orange curve).

After administration of ZEN (3 mg/kg BW) to pigs, ZEN-GlcA was chosen as biomarker in plasma and urine (Figure 5). The best biomarker in dried feces was ZEN (Figure 6). The maximum concentration for ZEN-GlcA in plasma was achieved after 20 min. In urine the maximum concentration was obtained between 4–8 h. In dried feces the concentration of ZEN may still be rising after 24 h. Also, for ZEN, no difference in mycotoxin concentration could be observed between the two groups. 

In Table 4 an overview is given of the determined toxicokinetic parameters. 

## 3. Discussion

### 3.1. Biomarkers for Exposure

In this study, the efficacy of a mycotoxin detoxifier was determined by monitoring biomarkers in animal biological fluids. Therefore, possible biomarkers were determined using a targeted LC-MS/MS and LC-HRMS approach. 

#### 3.1.1. Broiler Chickens

After administration of AFB1 to broiler chickens, AFB1 and AFL were detected in plasma. The presence of AFL in plasma is in accordance with an in vitro analysis performed by Lozano and Diaz [39]. These authors showed that AFL and AFBO were the two most prevalent metabolites after incubation of AFB1 with the microsomal and cytosolic parts of hepatocytes of different poultry species (turkeys, broiler chickens, quails, and ducks). AFBO was not detected in this study since AFBO is mainly bound to albumin in plasma [40]. The albumin-AFBO complex could not be retrieved here because albumin/lysine adducts were precipitated during the sample preparation, required for the subsequent mass spectrometric analysis. This poses no problem since AFB1-lysine is a biomarker for chronic exposure and AFB1 is a good biomarker for acute exposure, which is important when testing the efficacy of a detoxifier using the short-term in vivo model in this study. In humans, appropriate markers for dietary exposure in serum are AFB1-albumine, AFB1-lysine, and AFB1 itself [41]. In the present study, AFB1 showed the highest peak area and was selected as the optimal biomarker for measuring the efficacy of the mycotoxin detoxifier in plasma. In the excreta samples, only AFB1 was detected. AFB1 as a major metabolite in excreta has also been confirmed by Cortés et al. who analyzed the litter of poultry fed diets contaminated with AFB1 [42]. 

In this study, OTA was the main component identified in broiler chicken plasma and excreta after administration of OTA. Ochratoxin A metabolites were searched for in excreta and plasma of broiler chickens using HR-MS (untargeted approach). These (unknown) metabolites either were not present or were present only in trace amounts. This confirms literature reports stating that biotransformation of OTA is limited [23]. A second peak was observed in the plasma-concentration time curve at 4 h administration p.a., coinciding with the moment of feeding. This could be considered as an indication of enterohepatic circulation of OTA. A similar trend was reported by Ringot et al. [24] and Devreese et al. [43] who proposed a biliary excretion and reabsorption of OTA. 

The low oral bioavailability and efficient biotransformation and excretion of DON in broiler chickens results in concentrations of DON and DOM-1 below the LOQ in blood [27,31]. Also, in excreta and chyme samples no traces of DOM-1 could be detected after administration of DON [44]. Consequently, DON and DOM-1 are not considered ideal biomarkers in broiler chicken plasma and excreta. On the other hand, the most abundant metabolite in these samples is DON-S, as demonstrated in the present and previous studies [27,31,44]. Since separation on the HRMS instrument was not achieved between DON-3-sulphate and DON-15-sulphate, the presence of DON-15-sulphate could not be completely eliminated although its presence is considered highly unlikely [44]. Consequently, DON-sulphate forms the point of discussion in this study instead of DON-3-sulphate. 

#### 3.1.2. Pigs

In pigs, no biomarkers could be identified in feces after administration of DON. High absorption of DON has previously been reported in the small intestine which leads to low level (2–3%) excretion via feces [45,46]. The mycotoxins detected here in feces were below the limit of quantification and could thus not be considered biomarkers. The primary excretion route for DON is via urine [47] which was also confirmed here. Therefore, DON itself proved the best biomarker for this matrix. The highest concentration of DON in urine tends to be between 4 to 8h after exposure [46] which was also confirmed in our study. Additionally, DON-GlcA was detected in urine but to a lesser extent (below LOQ) which is also in agreement with literature [46]. In this study, pig plasma contained DON-GlcA as the most prevalent metabolite and was considered thus, the best biomarker for this matrix. Furthermore, low amounts of DON were detected in plasma. Both findings about levels of DON-GlcA and DON in plasma after administration of DON to pigs are in full agreement with previous studies [31]. In conclusion, DON in urine and DON-GlcA in plasma are considered appropriate biomarkers for short term exposure in pigs. 

Only ZEN and ZEN-GlcA but no other metabolites were detected in pig plasma in this study upon oral administration of ZEN which is in full agreement with De Baere et al. [48]. ZEN-GlcA was considered the optimal biomarker in pig plasma whereas, ZEN in pig dried feces was selected with its largest amount being excreted after 24 h. This late excretion can be attributed to the enterohepatic circulation of ZEN. Similar results were observed by Binder et al. who reported excretion of ZEN and α-ZEL in lyophilized feces after administration of ZEN [49]. In pig urine the most abundant molecules were ZEN and ZEN-GlcA, excreted primarily within the first 24 h and, ZEN-GlcA was considered the optimal biomarker. Similarly, Binder et al. [49] observed that the largest part of the administered dose of ZEN was excreted into urine as ZEN-GlcA within the first 24 h after application. 

In this study the exact position of the glucuronide group in both molecules could not be defined. Therefore, only a generic term was used, i.e., DON-GlcA and ZEN-GlcA. 

### 3.2. Efficacy of the Mycotoxin Detoxifier

#### 3.2.1. Broiler Chickens

No statistically significant difference was observed between the two groups for OTA in broiler chicken plasma and excreta, which indicates no effect of the detoxifier used here. Although not directly comparable due to differences in composition, Khatoon et al. [3] and Bhatti et al. [11] reported no influence of the OTA-induced immunosuppressive effects upon addition of a plain toxin binder (sodium bentonite) to the feed in broiler chickens. 

In contrast to OTA, the mycotoxin detoxifier significantly reduced systemic exposure to AFB1 and DON. More specifically, this study showed a reduced concentration of DON in broiler plasma upon administration of the candidate mycotoxin detoxifier. These findings are especially important taken into account the direct toxicity of DON and its predisposing role in necrotic enteritis in chickens as well as in a variety of pathogens and their related diseases in different animal species [50]. Similarly, reduction in broiler plasma concentration was also observed for AFB1 upon administration of the candidate mycotoxin detoxifier. This is perhaps not of surprise as most common detoxifiers based on a bentonite clay component (or variations of it) have the capacity to bind to the polar AFB1 [12,51] and it is believed that the same action has been exerted here by the clay components of the mycotoxin detoxifier under test. In fact, bentonite is registered by EFSA as an effective binder for AFB1 [52]. 

The positive effect of the detoxifier on the combined DON and AFB1 contamination could be attributed to the extra components in the formulation besides clay, i.e., yeast cell wall extracts and a blend of plant extracts amongst others. The detoxifier tested here claims several modes of action: binding/adsorption of the mycotoxins in the intestines of the animals, activation and supporting the liver function and thus the detoxification process in general, as well as enhancement of the immune system (unpublished data). 

Due to limitations of the adopted sampling strategy it was not feasible to evaluate the impact of the mycotoxin detoxifier in excreta. Namely, samples were only collected at selected time points and not continuously, leading to low number of data with increased variability in reported concentrations. 

#### 3.2.2. Pigs

In pigs, no difference in AUC of DON or ZEN in all matrices studied was seen between the control and treatment groups. However, it should be noted that the dose of the detoxifier (1 g/kg feed) was retrospectively regarded too low in relation to the very high concentration of these two mycotoxins in pigs. For example, Jiang et al. showed a dose dependent relationship between the dose of a bentonite-based binder and the detoxifying effect. The lower concentrations of the mycotoxin detoxifier (1 and 2 g/kg feed) only partially reduced the effects caused by 1 mg/kg of ZEN contaminated feed [53]. However, in this study although the dose of detoxifier also corresponded to approximately 1 g/kg feed, the dose of ZEN was 75 times higher (i.e., ~75 mg/kg feed) than that applied in the study of Jiang et al. and 300 times higher than the European guidelines (0.1 mg/kg feed) for feedingstuff in pigs. It is believed that this high dose of ZEN could explain the lack of efficacy of this mycotoxin detoxifier during the simultaneous administration of ZEN and DON in pigs. 

The effects of binders containing bentonite on the symptoms of DON intoxication in literature are variable. Jin et al. showed an increase in growth and feed intake when adding the binder (1 kg/ton feed) to the contaminated diet [54]. Positive results were also obtained by Shehata et al., who observed increasing concentrations of DON in urine after adding the binder [55]. However, these positive findings could not be reproduced by Frobose et al. and Döll et al. [56,57]. In the present study, no effects of the detoxifier on DON absorption in pigs were observed. However, the difference in efficacy of the detoxifier between pigs and broiler chickens for DON could mainly be related to a) the much higher concurrent dose of ZEN in the case of pigs and b) to the increased ratio of mycotoxins to detoxifier i.e., the ratio of mycotoxins to detoxifier (3/100) in pigs was 3 times higher than that in chickens (1/100). 

In conclusion, this proof-of-concept study demonstrated the efficacy in broiler chickens for DON and AFB1 of a candidate mycotoxin detoxifier after simultaneous administration of blends of mycotoxins using the proposed biomarkers for exposure in different biological matrices. Future research is needed to investigate if these biomarkers for exposure can be correlated with the health status of the animal. The detoxifier used in this study has shown great promise in reducing the systemic absorption of AFB1 and DON in broiler chickens, although further experiments should be done to include other ratios of mycotoxins to detoxifier dose. 

## 4. Material and Methods

### 4.1. Chemicals, Products, and Reagent

The analytical standards of ZEN, OTA, AFB1, AFM1, DON, 3-acetyldeoxynivalenol (3ADON), abd 15-acetyldeoxynivalenol (15ADON) were obtained from Fermentek (Jerusalem, Israel). Zearalanone (ZAN), AZEL, BZEL, AZAL, and BZAL were purchased from Sigma-Aldrich (Bornem, Belgium), while DOM-1 was obtained from Biopure (Tulln, Austria). Internal standards (IS) ^13^C_15_-DON, ^13^C_18_-ZEN, ^13^C_20_-OTA, and ^13^C_17_-AFB1 were purchased from Biopure (Tulln, Austria). All standards were stored at ≤−15 °C. 

Water, methanol (MeOH) and acetonitrile (ACN) were of LC-MS grade and were obtained from Biosolve (Valkenswaard, The Netherlands). Ammonium formate, glacial acetic acid, formic acid and ethyl acetate were of analytical grade and were purchased from VWR (Leuven, Belgium). Millex^®^-LG filter units (0.2 µm), sodium hydroxide pellets, hydrochloric acid 37% fuming solution, acetone, and ethanol of analytical grade were obtained from Merck (Overijse, Belgium). Dimethyl sulfoxide (DMSO) was purchased from Sigma-Aldrich (Bornem, Belgium). Ostro^®^-96 well plates were obtained from Waters (Zellik, Belgium). HybridSPE^®^—phospholipid 30 mg/1 mL solid phase extraction (SPE) tubes were purchased from Sigma-Aldrich (Overijse, Belgium). 

DON and OTA for the animal trials were dissolved in analytical grade ethanol, and AFB1 and ZEN in DMSO to obtain stock solutions of 10 mg/mL DON, 5 mg/mL OTA, 30 mg/mL ZEN, and 10 mg/mL AFB1. The solutions of DON, OTA, and AFB1 were combined and mixed with HPLC-grade water to obtain a solution per broiler chicken with a concentration of 0.5 mg/kg BW DON, 0.25 mg/kg BW OTA and 2 mg/kg BW AFB1. For pigs, the solutions of DON and ZEN were combined and mixed with HPLC-grade water to obtain a solution with a concentration of 36 µg/kg BW DON and 3 mg/kg BW ZEN that was administered to the pigs. 

The standard stock solutions used for chromatographic analysis were prepared in ACN. The concentration was 100 µg/mL for ZEN, AZAL, BZAL, AZEL, BZEL, ZAN, DON, AFB1, and AFM1 and 10 µg/mL for OTA. The remaining standards were purchased as solutions, i.e., 3ADON (100 µg/mL in ACN), 15ADON (100 µg/mL in ACN) and DOM-1 (50 µg/mL in ACN). A standard stock solution of 10 µg/mL in ACN was prepared for DOM-1. A combined working solution of all analytical standards (without internal standard (IS)) at a concentration of 1 µg/mL was prepared. Serial dilutions of the combined working solutions were prepared yielding working solutions with concentrations of 100 ng/mL and 10 ng/mL. 

All internal standards (IS) were obtained as solutions, i.e., ^13^C_15_-DON (25 µg/mL in ACN), ^13^C_20_-OTA (10 µg/mL in ACN), ^13^C_18_-ZEN (25 µg/mL in ACN), and ^13^C_17_-AFB1 (0.5 µg/mL in ACN). Individual working solutions of 1 µg/mL were made for all IS, except for ^13^C_17_-AFB1 (100 ng/mL). Next, a combined working solution of all IS was prepared with a final concentration of 100 ng/mL for all components, except ^13^C_17_-AFB1 (10 ng/mL). All working solutions were stored at ≤−15 °C. 

### 4.2. Broiler Chicken Trial

A total of sixteen healthy broiler chickens (Ross 308, 3 weeks of age, 1.05 ± 0.11 kg, ♂/♀, 5/11) were obtained from a commercial farm in Horebeke, Belgium. They were randomly allocated in two groups of 8 chickens. Each group was housed in a pen of 4 m^2^ with a bedding of wood shavings. In each pen a perch was at the animals’ disposal. The lighting program was 18 h of light and 6 h of darkness. The temperature was kept between 18 and 25 °C, if needed a heating lamp per pen was available. The animals were housed under these conditions for one week to acclimatize. Water and control feed were provided ad libitum during this period. 

Feed (Farm 2 pure mix) was purchased from Versele-Laga/Quartes (Deinze, Belgium). This control feed was analyzed for the presence of mycotoxins by Primoris (Zwijnaarde, Belgium). The following mycotoxins were analyzed using LC-MS/MS: 3- and 15ADON, aflatoxins B1, B2, G1, G2, cytochalasin E, DON, FB1 and FB2, nivalenol, OTA, T-2/HT-2 toxin and ZEN. The control feed contained 143 µg/kg of DON and 20 µg/kg of ZEN. 

After the one-week acclimatization period (day 8), feed was withheld for 12 h before the start of the trial, until 4 h after administration. The first group of eight broiler chickens (control group) received a single intra-crop bolus of mycotoxins containing 0.5 mg/kg BW DON, 0.25 mg/kg BW OTA and 2 mg/kg BW AFB1. This corresponds with a concentration of 5 mg DON, 2.5 mg OTA and 20 mg AFB1 per kg feed. The relationship with the European limits in feed is shown in supplementary Table S13. These higher doses are needed in order to obtain sufficient plasma levels of the mycotoxins, due to specific toxicokinetic characteristics of the mycotoxins tested. These doses result in satisfactory and reproducible plasma concentration–time profiles. The most important read-out of the short-term in vivo efficacy model used in this study is the area under the plasma concentration–time curve (AUC), this has to be high enough to be able to demonstrate a statistically significant reduction in AUC when combined with the detoxifier. 

The same bolus of mycotoxins was administered to the second group of eight broiler chickens (treatment group). Additionally, these birds received immediately after the mycotoxins an intra-crop bolus of a candidate mycotoxin detoxifier at a dose of 0.237 g/kg BW, corresponding to 2.4 kg per ton feed. The mycotoxin detoxifier tested is a proprietary commercial product (Escent^®^ S) consisting of a binder component (i.e., bentonite, sepiolite), preservatives and mold inhibitors, yeast cell wall components and a proprietary blend of plant extracts rich in natural polyphenols and antioxidants aimed to support the animal’s natural detoxifying processes (Innovad SA, Essen, Belgium). The ratio of total amount of mycotoxins per kg BW to the dose of binder per kg BW was 2.75 mg/237 mg or ±1/100. Tap water was added to the product to form a suspension for administration. To adjust for the administration of the binder, the first group received the same volume of tap water. Additionally, a bolus of tap water was administered to both groups in order to flush the tube and crop. Next, 1 mL of blood was collected via the *vena metatarsalis plantaris superficialis* before administration (blank sample) and at 0.08, 0.16, 0.33, 0.5, 0.75, 1, 1.5, 2, 3, 4, 6, 10, 12, 24, 36, and 48 h post-administration (p.a.) in heparinized tubes (Vacutest Kima, Novolab, Geraardsbergen, Belgium). The samples were centrifuged (3724 *g*, 10 min, 4 °C) and plasma was stored at ≤15 °C until analysis. Also, excreta samples were taken at different time points from each bird separately by placing each animal in a box at the different time points: before administration (blank sample) and at 2, 4, 6, 8, 10, 12, 24, 36, and 48 h p.a. The excreta samples were lyophilized and stored at ≤15 °C until analysis. 

### 4.3. Pig Trial

A total of eight hybrid pigs (6 weeks of age, 9.94 ± 1.24 kg BW, ♂/♀, 4/4) were obtained from the Research Institute for Agriculture, Fisheries and Food (ILVO, Melle, Belgium). The animals were randomly allocated to two groups of four pigs. Each group was housed in a compartment of 4 m^2^ in the barn. In each compartment, toys were at the animals’ disposal and were changed regularly to avoid boredom. The lighting was natural by windows in the barn. The temperature was kept between 22–26 °C. The animals were housed under these conditions for one week to acclimatize. Water and control feed were provided ad libitum during this period. This study was a cross-over study with a wash-out period of two days. 

Feed (Biggistart Opti) was purchased from Aveve (Leuven, Belgium). This control feed was also analyzed for the presence of mycotoxins by Primoris (Zwijnaarde, Belgium). The same mycotoxins were analyzed as for the chicken feed. The control feed contained 139 µg/kg of DON and 12 µg/kg of ZEN. 

After the one-week acclimatization period (day 8), feed was withheld for 12 h before the start of the trial, until 4 h p.a. The first group of four pigs (control group) received an intra-gastric bolus of mycotoxins containing 36 µg/kg BW DON and 3 mg/kg BW ZEN, corresponding with 0.9 mg DON and 75 mg ZEN per kg feed. The relationship with the European limits in feed is shown in supplementary Table S13. The same bolus of mycotoxins was administered to the second group of four pigs (treatment group). Additionally, these pigs received an intra-gastric bolus of the candidate mycotoxin binder at a dose of 0.1 g/kg BW or 1 kg per ton feed. The mycotoxin detoxifier used was the same as described in paragraph 2.2. The ratio of total amount of mycotoxins per kg BW to the dose of binder per kg BW was 3.036 mg/100 mg or ±3/100. Tap water was added to the product to form a suspension for administration. To adjust for the administration of the binder, the first group received the same volume of water. Additionally, a bolus of tap water was administered to both groups in order to flush the tube. 

Next, 3 mL of blood was collected via the *vena jugularis* before administration (blank plasma) and 0.08, 0.16, 0.33, 0.5, 0.75, 1, 1.5, 2, 3, 4, 6, 10, 12, and 24 h p.a. in heparinized tubes (Vacutest Kima, Novolab, Geraardsbergen, Belgium). The samples were centrifuged (3724 *g*, 10 min, 4 °C) and plasma was stored at ≤−15 °C. Next, also individual feces were sampled from each pig by rectal stimulation at different time points: before administration (blank sample) and 2, 4, 6, 8, 10, 12, and 24 h p.a. The feces samples were lyophilized and stored at ≤−15 °C until analysis. Finally, also urine was sampled using pediatric urine collection bags. These were applied to the male pigs only, as described by *Gasthuys* et al. [58]. Samples were taken before administration (blank sample) and at 4 intervals: 0–4 h, 4–8 h, 8–12 h, and 12–24 h p.a. 

**Figure 7:**
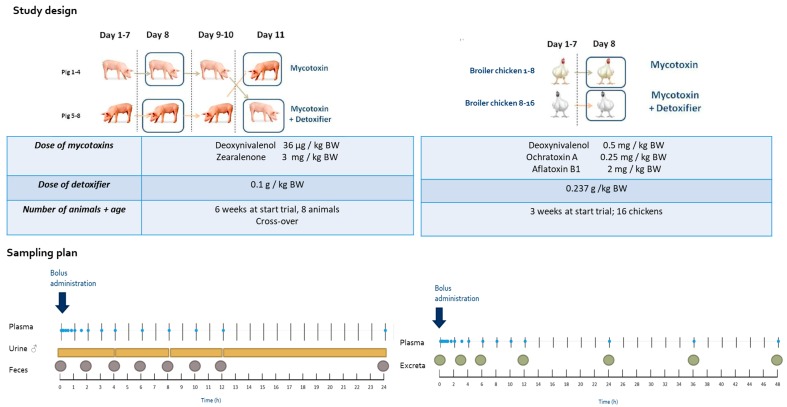
Flowchart of the animal trials.

All animal experiments were approved by the ethical committee of the Faculty of Veterinary Medicine and the Faculty of Bioscience Engineering of Ghent University (EC2017/05) on 30 March 2017. A flowchart summarizing the animal trials is shown in Figure 7. 

### 4.4. Sample Preparation, LC-MS/MS and LC-HRMS Analysis

Sample pretreatment and LC-MS/MS and HRMS analysis were performed as previously described [18]. 

In brief, 150 µL of chicken plasma was brought onto a well of an Ostro^®^-plate and 15 µL of a 100 ng/mL (except ^13^C_17_-AFB1 10 ng/mL) IS combined working solution and 450 µL of ACN with 0.1% formic acid were added. The Ostro^®^ plate was brought under vacuum and the eluate was dried under a gentle N_2_ stream at 40 ± 5 °C and reconstituted in 150 µL of MeOH/water (85/15; *v*/*v*). An aliquot of 5 µL was injected onto the chromatographic instruments. The lyophilized excreta were equally divided (250 mg/tube) over two tubes. Next, 20 µL of a 100 ng/mL (except ^13^C_17_-AFB1 10 ng/mL) IS combined working solution and 1.5 mL of ACN were added. Then, 250 µL of 1 M HCl were added to the first tube. The two tubes were vortex mixed and shaken during 15 min on a vertical rotator, followed by centrifugation (3724 *g*, 10 min, 4 °C). The supernatant was dried under N_2_ at 40 ± 5 °C. The dried samples were reconstituted in 250 µL of MeOH/water (85/15; *v*/*v*) and filtered through a Millex^®^-LG 0.2 µm filter into a vial. An aliquot of 5 µL was injected onto the LC-MS/MS and LC-HRMS instrument. 

To 250 µL of pig plasma, 20 µL of a 100 ng/mL (except ^13^C_17_-AFB1 10 ng/mL) IS combined working solution and 750 µL of ACN with 0.1% formic acid were added, followed by vortex mixing (10 s) and centrifugation (8517 *g*, 10 min, 4 °C). The supernatant was collected and dried under a N_2_-stream at 40 ± 5 °C. The dried supernatant was reconstituted in 250 µL of MeOH/water (85/15; *v*/*v*), followed by vortex mixing. The reconstituted sample was transferred into an autosampler vial and an aliquot (5 µL) was injected onto the LC-MS/MS and LC-HRMS. Five hundred µL of urine were mixed with 20 µL of a 100 ng/mL (except ^13^C_17_-AFB1 10 ng/mL) combined IS working solution. The pH was determined by means of pH test strips and was adjusted to pH 8 using a 0.1 M NaOH solution. Three mL of ethyl acetate were added to each tube, followed by vortex mixing for 10 s and rotating during 15 min on a horizontal roller shaker (Staffordshire, UK). Finally, the tubes were centrifuged for 10 min at 3724 *g* and 4 °C. The organic phase was collected and evaporated to dryness using a gentle N_2_-stream at 40 ± 5 °C. The dried sample was reconstituted in 250 µL of MeOH/water (85/15; *v*/*v*) and after vortex mixing added to an autosampler vial. An aliquot (5 µL) was injected onto the equipment. Twenty µL of a 100 ng/mL (except ^13^C_17_-AFB1 10 ng/mL) IS combined working solution and 5 mL of acetone were added to a tube containing 250 mg freeze dried feces. The tube was shaken for 40 min on a vertical rotator, followed by centrifugation (3724 *g*, 10 min, 4 °C). The supernatant was brought onto HybridSPE-phospholipid cartridges. The eluate was evaporated until dry using a gentle N_2_-stream at 40 ± 5 °C. The dried sample was reconstituted in 250 µL of MeOH/water (85/15; *v*/*v*) and filtered through a Millex^®^-LG 0.2 µm filter into a vial. An aliquot (5 µL) was injected onto the LC-MS/MS and LC-HRMS instrument. 

The UHPLC-MS/MS system consisted of an Acquity H-Class UPLC coupled to a Xevo TQ-S mass spectrometer. The same type of UPLC pump was coupled to a Synapt^®^ G2-Si high definition mass spectrometer (Waters, Zellik, Belgium). Chromatographic separation was achieved on an HSS T3 column (100 mm × 2.1 mm i.d., dp: 1.8 μm) and a guard column of the same type (5 mm × 2.1 mm i.d., dp: 1.8 μm), both from Waters (Zellik, Belgium). The temperatures of the column oven and autosampler tray were set a 45 °C and 8 °C, respectively. A gradient elution program was run in the positive ionization mode with mobile phases (MP) of 10 mM ammonium formate and 0.3% formic acid in water (MP A), and 10 mM ammonium formate and 0.3% formic acid in MeOH (MP B). For the negative ionization mode, 1% acetic acid in water (MP C) and 1% acetic acid in ACN (MP D) were used. A gradient elution program was run for each ionization mode separately: for ESI positive: 0–1.5 min, 95% A, 5% B; 1.5–3 min, linear gradient to 40% A; 3–5 min, 40% A, 60% B; 5.0–10 min, linear gradient to 20% A; 10–10.50 min, linear gradient to 1% A; 10.50–13.0 min, 1% A, 99% B; 13–14 min, linear gradient 95% A; 14.0–16.0 min, 95% A, 5% B. For ESI negative: 0–1.5 min, 95% C, 5% D; 1.5–3 min, linear gradient to 60% C; 3.0–4.0 min, 60% C, 40% D; 4.0–7.0 min, linear gradient to 40% C; 7.0–9.0 min, 40% C, 60% D; 9.0–9.5 min, linear gradient 95% C; 9.5–12.0 min, 95% C, 5% D. The flow rate was set at 300 μL/min. The chromatographic parameters were the same for both MS instruments. 

The Xevo TQ-S mass spectrometer operated in selected reaction monitoring mode (SRM). For every compound, the two most intense product ions were selected for quantification and qualification, respectively. The settings on the Xevo^®^ TQ-S mass spectrometer were as follows: desolvation gas flow rate: 800 L/h, desolvation temperature: 600 °C, cone gas flow rate: 150 L/h, source temperature: 150 °C. The capillary voltage was optimized at 3.2 kV for ESI positive and 3.0 kV for ESI negative mode, respectively. Dwell times of 25 and 10 msec/transition were selected for each component separately. Table S1 shows the precursor ion, product ions (qualifier and quantifier) and main instrument settings for the different mycotoxins. The method was validated in compliance with the recommendations and guidelines defined by the European and international community [59,60,61]. Following parameters were evaluated: linearity, within-day and between-day precision and accuracy, limit of quantification (LOQ), limit of detection (LOD), carry over, specificity, recovery and signal suppression and enhancement (SSE). Limits of quantification were 1 ng/mL for most components, with a few exceptions ranging to 5 ng/mL. The limits of detection were between 0.001 and 1.68 ng/mL. Full validation results are described by *Lauwers* et al. [18] and are shown in supplementary Tables S2–S11. 

The Synapt G2-Si HDMS acquisition was performed using MS^E^ continuum scan function. The settings were as follows: low mass, 50 Da; high mass, 1200 Da; scan time, 0.1 s; data format, continuum. The lock mass solution consisted of leucine encephalin (200 pg/µL). The lock spray was acquired during HRMS acquisition, but not corrected. The lock spray correction (*m/z* 556.276575; *m/z* 554.26202) and data processing was performed using Unify 1.8 software (Waters, Zellik, Belgium). For every mycotoxin and some of their phase I and II metabolites, the accurate masses [M] were defined as shown in supplementary Table S12. In the software, additional adducts (Na^+^, NH_4_^+^, CH_3_COO^−^, HCOO^−^) and transformations (glucuronidation, di-glucuronidation, sulphatation, oxidation, glutathione conjugation) were selected. 

The mycotoxins for which standards were available (DON, DOM1, 3/15ADON, OTA, AFB1, AFM1, ZEN, AZEL, BZEL, AZAL, BZAL, and ZAN) were determined using LC-MS/MS. The method was fully validated, based on a quantitative and targeted approach. The mycotoxins for which no reference standards were available were determined using LC-HRMS and hence, this method was qualitative and untargeted. 

The most appropriate biomarkers for exposure were selected based on the highest HRMS or MS/MS instrument response expressed as peak areas. 

### 4.5. Toxicokinetic and Statistical Analysis

The following toxicokinetic parameters were determined: area under the concentration–time curve from time zero to the last point above LOQ or to infinity (AUC_0→t_ or AUC_0→__∞_, respectively), maximum concentration (C_max_) and time to maximum concentration (T_max_) using non-compartmental analysis (Phoenix, Princeton, NJ, USA). 

The AUC was used to evaluate the efficacy of the detoxifier. The detoxifier was considered effective when its respective AUC was significantly lower when compared to the AUC without detoxifier, in plasma and urine. In feces, the detoxifier was considered effective when the respective AUC of the toxin was significantly higher with than without detoxifier. The percentage difference in AUC_0→∞_ between treatment and control was calculated using the formula:

$$\frac{Treatment\;{AUC}_{0\to \infty }-Control\;{AUC}_{0\to \infty }}{Control\;{AUC}_{0\to \infty }}$$

The statistical analysis was done using SPSS via two-sample *t*-test. The level of significance was set at 0.05. 

## Figures and Tables

**Table 1:** Most relevant biomarkers for exposure according to European Food Safety Authority (EFSA) [17].

Mycotoxin(s) against Which the Additive Is Intended to Act	Most Relevant Biomarkers for Exposure
Aflatoxin B1 (AFB1)	Aflatoxin M1 in milk/egg yolk
Deoxynivalenol (DON)	DON/metabolites in blood serum
Zearalenone (ZEN)	ZEN + α- and β-zearalenol in plasma Excretion of ZEN/metabolites
Ochratoxin A (OTA)	OTA in kidney (or blood serum)
Fumonisins B1+B2	Sphinganine/sphingosine ratio in blood, plasma or tissue

**Table 2:** The mycotoxins and their metabolites detected in biological matrices (plasma, urine, and dried feces or plasma and dried excreta) after administration of DON (36 µg/kg BW) and ZEN (3 mg/kg BW) to pigs and DON (0.5 mg/kg BW), OTA (0.25 mg/kg BW) and AFB1 (2 mg/kg BW) to broiler chickens. The most appropriate biomarkers are indicated in bold. The limits of quantification (LOQ) of DON and DOM1 in pig feces was 5 ng/g. The LOQ for DON-GlcA was set at a peak area of 100/250 mg feces.

**Pig**	**Plasma**	**Urine**	**Feces**
DON	DON **DON-GlcA**	**DON**, DOM-1 DON-GlcA (below LOQ)	DON (below LOQ) DOM-1 (below LOQ)
ZEN	ZEN **ZEN-GlcA**	ZEN, AZEL, BZEL, ZAN **ZEN-GlcA**	**ZEN**, AZEL, BZEL, AZAL, BZAL, ZAN
**Broiler Chicken**	**Plasma**	**Excreta**	
AFB1	AFL **AFB1**	**AFB1**	
OTA	**OTA**	**OTA**	
DON	**DON-S**	**DON-S**	

DON (deoxynivalenol), DON-GlcA (deoxynivalenol-glucuronide), DOM-1 (de-epoxy-deoxynivalenol), ZEN (zearalenone), ZEN-GlcA (zearalenone-glucuronide), AZEL (α-zearalenol), BZEL (β-zearalenol), ZAN (zearalanone), AZAL (α-zearalanol), BZAL (β-zearalanol), AFB1 (aflatoxin B1), AFL (aflatoxicol), OTA (ochratoxin A) and DON-S (deoxynivalenol-sulphate).

**Table 3:** Mean toxicokinetic parameters determined after single oral administration of DON (0.5 mg/kg BW), OTA (0.25 mg/kg BW) and AFB1 (2 mg/kg BW) to broiler chickens, either with (treatment group, *n* = 8) or without detoxifier (control, *n* = 8).

Mycotoxins in Matrix	Treatment or Control	Area Under the Concentration–Time Curve from Time Zero to the Last Time Point (AUC_0→t_)	Area Under the Concentration–Time Curve from Time Zero to Infinity (AUC_0→__∞_)	% Difference of AUC_0→__∞_ between Treatment and Control [(Tr-C)/C)]	Maximum Concentration Cmax	Time to Maximum Concentration Tmax (h)
DON-S in plasma	Treatment	1,484,621 (peak area * h)	1,545,085 (peak area * h)	−49% *	26,260 (peak area)	0.25
DON-S in plasma	Control	2,960,870 (peak area * h)	3,009,746 (peak area * h)		52,183 (peak area)	0.5
DON-S in excreta	Treatment	n.a.	n.a.		118,670 (peak area)	3.00
DON-S in excreta	Control	n.a.	n.a.		55,753 (peak area)	6.00
AFB1 in plasma	Treatment	992 (h * ng/mL)	1156 (h * ng/mL)	−40% *	11.5 ng/mL	0.50
AFB1 in plasma	Control	1600 (h * ng/mL)	1914 (h * ng/mL)		9.8 ng/mL	0.25
AFB1 in excreta	Treatment	n.a.	n.a.		3309 ng/g	6.00
AFB1 in excreta	Control	n.a.	n.a.		2400 ng/g	6.00
OTA in plasma	Treatment	16,874 (h * ng/mL)	17,560 (h * ng/mL)	+13%	70.3 ng/mL	0.25
OTA in plasma	Control	16,315 (h * ng/mL)	17,322 (h * ng/mL)		62.1 ng/mL	0.083
OTA in excreta	Treatment	n.a.	n.a.		8252 ng/g	3.00
OTA in excreta	Control	n.a.	n.a.		5838 ng/g	6.00

N.a.: not applicable due to the limited number of samples. * *p* < 0.05.

**Table 4:** Mean toxicokinetic parameters determined after single oral administration of DON (36 µg/kg BW) and ZEN (3 mg/kg BW) to pigs, either with (treatment group, *n* = 8) or without detoxifier (control, *n* = 8).

Mycotoxins in Matrix	Treatment or Control	Area Under the Concentration–Time Curve from Time Zero to the Last Sampling Point (AUC_0→t_)	Area Under the Concentration–Time Curve from Time Zero to Infinity (AUC_0→__∞_)	% Difference of AUC_0→__∞_ between Treatment and Control [(Tr-C)/C)]	Maximum Concentration Cmax	Time at Maximum Concentration Tmax (h)
DON-GlcA in plasma	Treatment	3287 (peak area * h)	4739 (peak area * h)	−13%	600 (peak area)	3
DON-GlcA in plasma	Control	4819 (peak area * h)	5433 (peak area * h)		755 (peak area)	4
DON in urine	Treatment	9729 (h * ng/mL)	12,429 (h * ng/mL)	−26%	2532 ng/mL	4–8
DON in urine	Control	11,990 (h * ng/mL)	17,006 (h * ng/mL)		1597 ng/mL	4–8
ZEN-GlcA in plasma	Treatment	54,606 (peak area * h)	56,594 (peak area * h)	−12%	12,247 (peak area)	0.75
ZEN-GlcA in plasma	Control	61,708 (peak area * h)	64,456 (peak area * h)		19,487 (peak area)	0.33
ZEN-GlcA in urine	Treatment	19,559,385 (peak area * h)	24,454,352 (peak area * h)	−4%	1,640,555 (peak area)	4–8
ZEN-GlcA in urine	Control	16,850,362 (peak area * h)	25,470,498 (peak area * h)		1,639,248 (peak area)	4–8
ZEN in feces	Treatment	267,115 (h * ng/g)	n.a.	+21%	n.a.	n.a.
ZEN in feces	Control	220,788 (h * ng/g)	n.a.		18,621 ng/g	12

N.a.: not applicable since Cmax was not yet reached. * *p* < 0.05.

## Supplementary Materials

The following are available online at https://www.mdpi.com/2072-6651/11/4/187/s1, Table S1: Mycotoxins, measured form/adduct, molecular mass, product ions, cone voltage, collision energy and retention time used for UHPLC-MS/MS analysis, Table S2: Validation results for linearity (linear range, correlation coefficient (r) and goodness-of-fit coefficient (g)), limit of quantification (LOQ), and limit of detection (LOD) of 24 mycotoxins in pig feces, Table S3: Validation results for linearity (linear range, correlation coefficient (r) and goodness-of-fit coefficient (g)), limit of quantification (LOQ), and limit of detection (LOD) of 24 mycotoxins in pig urine, Table S4: Validation results for linearity (linear range, correlation coefficient (r) and goodness-of-fit coefficient (g)), limit of quantification (LOQ), and limit of detection (LOD) of 24 mycotoxins in broiler chicken plasma, Table S5: Validation results for linearity (linear range, correlation coefficient (r) and goodness-of-fit coefficient (g)), limit of quantification (LOQ), and limit of detection (LOD) of 25 mycotoxins in broiler chicken excreta, Table S6: Validation results for linearity (linear range, correlation coefficient (r) and goodness-of-fit coefficient (g)), limit of quantification (LOQ), and limit of detection (LOD) of 24 mycotoxins in pig plasma, Table S7: Results of the within-day and between-day precision and accuracy experiments for 24 mycotoxins in pig plasma, Table S8: Results of the within-day and between-day precision and accuracy experiments for 24 mycotoxins in pig feces, Table S9: Results of the within-day and between-day precision and accuracy experiments for 24 mycotoxins in pig urine, Table S10: Results of the within-day and between-day precision and accuracy experiments for 24 mycotoxins in broiler chicken plasma, Table S11: Results of the within-day and between-day precision and accuracy experiments for 25 mycotoxins in broiler chicken excreta, Table S12: Mycotoxins, phase I and II and other metabolites and accurate mass used for UHPLC-HRMS analysis, Table S13: Relationship between European limits and the dose administered.
