# Supplementary material for: Biomarkers for Exposure as a Tool for Efficacy Testing of a Mycotoxin Detoxifier in Broiler Chickens and Pigs
Source: Toxins (Basel). 2019 Mar 28;11(4):187. doi: 10.3390/toxins11040187 (PMC6520943; doi:10.3390/toxins11040187)
Supplement: Supplementary file 1 [file toxins-11-00187-s001.pdf]

Supplementary Materials: Biomarkers for Exposure as A Tool for Efficacy Testing of A Mycotoxin Detoxifier in Broiler Chickens and Pigs

Marianne Lauwers, Siska Croubels, Ben Letor, Christos Gougoulias and Mathias Devreese

Table S1. Mycotoxins, measured form/adduct, molecular mass, product ions, cone voltage, collision energy and retention time used for UHPLC-MS/MS analysis.

**ElectroSpray Ionisation (ESI) Positive Mode**							
**Compound**	**Measured Form/ Adduct**	**Precursor Ion (*m*/*z*)**	**Quantifier Ion (*m*/*z*)**	**Qualifier Ion (*m*/*z*)**	**Cone Voltage (V)**	**Collision Energy (eV)**	**Retention Time (min)**
Deoxynivalenol	[M+H$^+$]	297.0	249.1	203.4	20	9-14	4.36
De-epoxy-deoxynivalenol	[M+H$^+$]	281.0	215.0	233.0	30	12-12	4.70
3/15-acetyldeoxynivalenol	[M+H$^+$]	339.2	213.1	230.9	25	12-8	5.02
Aflatoxin M1	[M+H$^+$]	328.9	272.9	229.0	30	20-35	5.18
Aflatoxin B1	[M+H$^+$]	313.0	285.1	241.1	35	23-34	5.63
Ochratoxin A	[M+H$^+$]	404.0	238.9	220.8	35	20-32	9.23
[$^{13}C_{15}$]-Deoxynivalenol	[M+H$^+$]	312.0	263.0	245.0	20	10-10	4.37
[$^{13}C_{17}$]-Aflatoxin B1	[M+H$^+$]	330.10	255.1	301.0	20	35-28	5.63
[$^{13}C_{20}$]-Ochratoxin A	[M+H$^+$]	424.0	250.0	377.1	20	25-15	9.23
**ElectroSpray Ionisation (ESI) Negative Mode**							
**Compound**	**Measured Form/ Adduct**	**Precursor Ion (m/z)**	**Quantifier (m/z)**	**Qualifier (m/z)**	**Cone Voltage (V)**	**Collision Energy (eV)**	**Retention Time (min)**
zearalanone	[M-H]$^-$	319.1	275.0	205.0	20	20-22	8.41
α-zearalenol	[M-H]$^-$	319.2	275.0	301.0	20	20-22	6.87
α-zearalanol	[M-H]$^-$	321.2	277.2	303.3	30	23-20	6.75
β-zearalenol	[M-H]$^-$	319.2	275.0	301.0	20	20-22	7.51
β-zearalanol	[M-H]$^-$	321.2	277.2	303.3	30	23-20	7.36
zearalenone	[M-H]$^-$	317.1	175.0	130.8	15	25-30	8.50
[$^{13}C_{18}$]-Zearalenone	[M-H]$^-$	335.3	185.1	169.1	15	28-40	8.50

Table S2. Validation results for linearity (linear range, correlation coefficient (r) and goodness-of-fit coefficient (g)), limit of quantification (LOQ), and limit of detection (LOD) of 24 mycotoxins in pig faeces.

**Analyte**	**Linearity (*n* = 3 Different Days)**			**LOQ (ng/g)**	**LOD (ng/g)**
	**Linear Range (ng/g)**	**r ± SD**	**g (%) ± SD**		
ZEN	5–200	0.996 +/− 0.001	7.1 +/− 1.0	5.0	0.12
AZEL	1–200	0.996 +/− 0.001	15.7 +/− 4.3	1.0	0.42
AZAL	5–200	0.996 +/− 0.002	10.8 +/− 2.2	5.0	0.95
BZAL	1–200	0.996 +/− 0.002	9.0 +/− 3.5	1.0	0.47
BZEL	1–200	0.995 +/− 0.002	14.7 +/− 5.2	1.0	0.04
ZAN	1–200	0.997 +/− 0.003	11.5 +/− 4.2	1.0	0.16
TEA	1–200	0.999 +/− 0.000	18.7 +/− 0.9	1.0	0.21
AOH	5–200	0.997 +/− 0.001	8.9 +/− 3.1	5.0	1.10
AME	1–200	0.998 +/− 0.001	17.3 +/− 1.4	1.0	0.11
DON	5–200	0.994 +/− 0.003	14.1 +/− 4.4	5.0	0.41
DOM-1	5–200	0.997 +/− 0.000	8.8 +/− 3.1	5.0	1.68
3/15 ADON	1–200	0.998 +/− 0.001	13.4 +/− 4.2	1.0	0.44
T2	1–200	0.999 +/− 0.001	9.6 +/− 0.3	1.0	0.01
HT2	5–200	0.994 +/− 0.004	12.6 +/− 4.1	5.0	0.05
T2G	5–200	0.993 +/− 0.003	15.2+/− 5.3	5.0	0.02
AFB1	1–200	0.998 +/− 0.002	10.5 +/− 4.6	1.0	0.03
AFM1	1–200	0.998 +/− 0.000	7.9 +/− 4.2	1.0	0.03
OTA	1–200	0.997+/− 0.001	8.9 +/− 2.5	1.0	0.30
ENN A1	1–200	0.997 +/− 0.002	15.4 +/− 6.1	1.0	0.01
ENNA	1–100	0.996 +/− 0.003	12.6 +/− 7.0	1.0	0.15
ENNB	1–200	0.997 +/− 0.001	14.9 +/− 5.4	1.0	0.08
ENNB1	1–200	0.997 +/− 0.002	15.2 +/− 5.1	1.0	0.01
BEA	1–200	0.999 +/− 0.001	11.3 +/− 1.4	1.0	0.001

Table S3. Validation results for linearity (linear range, correlation coefficient (r) and goodness-of-fit coefficient (g)), limit of quantification (LOQ), and limit of detection (LOD) of 24 mycotoxins in pig urine.

Analyte	Linearity (*n* = 3 Different Days)			LOQ (ng/mL)	LOD (ng/mL)
	Linear Range (ng/mL)	r ± SD	g (%) ± SD		
ZEN	1-200	0.999 +/- 0.000	7.5 +/- 4.8	1.0	0.05
AZEL	1-200	0.998 +/- 0.001	10.8 +/- 7.0	1.0	0.05
AZAL	1-200	0.998 +/- 0.002	11.8 +/- 6.6	1.0	0.06
BZAL	1-200	0.999 +/- 0.000	6.3 +/- 3.0	1.0	0.07
BZEL	1-200	0.997 +/- 0.001	12.2 +/- 2.1	1.0	0.03
ZAN	1-200	0.998 +/- 0.001	13.1 +/- 5.2	1.0	0.04
TEA	1-200	0.998 +/- 0.002	11.0 +/- 4.6	1.0	0.04
AOH	1-200	0.995+/- 0.003	16.8 +/- 3.2	1.0	0.05
AME	1-200	0.997+/- 0.001	10.2 +/- 2.3	1.0	0.01
DON	1-200	0.998 +/- 0.001	9.3 +/- 1.1	1.0	0.02
DOM-1	4-200	0.995 +/- 0.004	9.3 +/- 5.8	4.0	0.86
3/15 ADON	1-200	0.998 +/- 0.001	11.9 +/- 5.0	1.0	0.08
T2	1-200	0.999 +/- 0.000	9.7 +/- 3.7	1.0	0.03
HT2	1-200	0.995 +/- 0.003	14.1 +/- 5.2	1.0	0.05
T2G	2-200	0.996 +/- 0.002	17.0 +/- 1.9	1.0	0.01
AFB1	1-200	0.999 +/- 0.000	5.1 +/- 4.2	1.0	0.01
AFM1	1-100	0.997 +/- 0.004	10.5 +/- 7.8	1.0	0.01
OTA	1-200	0.999 +/- 0.001	8.7 +/- 1.2	1.0	0.03
ENN A1	1-200	0.999 +/- 0.001	11.2 +/- 1.9	1.0	0.001
ENNA	1-200	0.998 +/- 0.002	7.6 +/- 4.6	1.0	0.05
ENNB	1-200	0.999 +/- 0.000	3.5 +/- 2.7	1.0	0.02
ENNB1	1-50	0.999 +/- 0.001	5.9 +/- 3.0	1.0	0.001
BEA	1-100	0.999 +/- 0.000	11.2 +/- 5.3	1.0	0.001

Table S4. Validation results for linearity (linear range, correlation coefficient (r) and goodness-of-fit coefficient (g)), limit of quantification (LOQ), and limit of detection (LOD) of 24 mycotoxins in broiler chicken plasma.

Analyte	Linearity (*n* = 3 Different Days)			LOQ (ng/mL)	LOD (ng/mL)
	Linear Range (ng/mL)	r ± SD	g (%) ± SD		
ZEN	1-200	0.998 +/- 0.001	8.0 +/- 6.4	1.0	0.18
AZEL	1-200	0.998 +/- 0.001	15.4 +/- 6.5	1.0	0.53
AZAL	1-200	0.997 +/- 0.003	12.0 +/- 5.2	1.0	0.10
BZAL	1-200	0.998 +/- 0.002	9.7 +/- 4.5	1.0	0.13
BZEL	1-200	0.998 +/-0.001	10.1 +/- 6.0	1.0	0.18
ZAN	1-200	0.998 +/-0.001	12.2 +/- 4.1	1.0	0.36
TEA	1-200	0.994+/-0.004	17.7 +/- 2.7	1.0	0.03
AOH	1-200	0.997 +/-0.002	13.6 +/- 5.8	1.0	0.23
AME	1-100	0.997 +/-0.002	15.6 +/- 5.6	1.0	0.01
DON	1-200	0.998 +/- 0.001	12.4 +/- 3.8	1.0	0.03
DOM-1	1-200	0.998 +/- 0.001	12.7 +/- 3.2	1.0	0.11
3/15 ADON	1-200	0.999 +/- 0.001	8.7 +/- 1.3	1.0	0.14
T2	1-200	0.997 +/- 0.003	7.7 +/- 3.7	1.0	0.01
HT2	1-200	0.999+/- 0.001	14.1+/- 8.0	1.0	0.05
T2G	5-200	0.996 +/- 0.004	9.0 +/- 5.0	5.0	0.05
AFB1	1-200	0.998+/- 0.002	6.3 +/- 4.3	1.0	0.01
AFM1	1-200	0.998 +/- 0.001	8.9+/- 5.0	1.0	0.01
OTA	1-200	0.998 +/- 0.002	12.7 +/- 3.5	1.0	0.43
ENN A1	1-200	0.999 +/- 0.000	7.9 +/- 1.0	1.0	0.001
ENNA	1-200	0.998+/- 0 .001	16.2 +/- 4.3	1.0	0.08
ENNB	1-100	0.999 +/- 0.000	5.9 +/- 3.1	1.0	0.001
ENNB1	1-200	0.997 +/- 0.002	14.0 +/- 2.2	1.0	0.001
BEA	1-200	0.999 +/- 0.001	8.9 +/- 5.4	1.0	0.001

Table S5. Validation results for linearity (linear range, correlation coefficient (r) and goodness-of-fit coefficient (g)), limit of quantification (LOQ), and limit of detection (LOD) of 24 mycotoxins in broiler chicken excreta.

Analyte	Linearity (*n* = 3 Different Days)			LOQ (ng/g)	LOD (ng/g)
	Linear Range (ng/g)	r ± SD	g (%) ± SD		
ZEN	1-200	0.998 +/- 0.001	14.8 +/- 3.1	1.0	0.001
AZEL	1-200	0.998 +/- 0 .001	13.2 +/- 4.8	1.0	0.21
AZAL	1-200	0.996 +/- 0.003	15.1 +/- 6.7	1.0	0.06
BZAL	1-200	0.997 +/- 0.002	13.9 +/- 4.7	1.0	0.06
BZEL	1-200	0.996 +/- 0.003	14.8 +/- 1.8	1.0	0.22
ZAN	1-200	0.999 +/- 0.001	13.7 +/-4.7	1.0	0.14
TEA	1-200	0.998 +/- 0.001	12.4 +/- 4.6	1.0	0.03
AOH	1-200	0.997 +/- 0.001	14.9 +/- 4.3	1.0	0.13
AME	1-200	0.998 +/-0.000	13.5 +/-1.6	1.0	0.06
DON	1-200	0.998 +/- 0.001	13.5 +/- 0.9	1.0	0.15
DOM-1	1-200	0.998 +/- 0.001	13.5 +/- 3.9	1.0	0.32
3/15 ADON	1-200	0.995 +/- 0.003	14.5 +/- 5.2	1.0	0.35
T2	1-200	0.999 +/- 0.000	5.1 +/- 0.2	1.0	0.03
HT2	4-100	0.997 +/-0.001	16.5 +/- 3.0	4.0	0.82
T2G	2-200	0.998 +/-0.000	13.6 +/- 5.3	2.0	0.77
AFB1	1-200	0.997 +/- 0.002	12.1 +/- 6.9	1.0	0.01
AFM1	1-200	0.998 +/- 0.002	12.2 +/- 3.1	1.0	0.001
OTA	1-200	0.998 +/- 0.001	11.4 +/- 5.0	1.0	0.12
ENN A1	1-200	0.995 +/- 0.004	16.8 +/- 4.9	1.0	0.01
ENNA	1-200	0.995 +/- 0.003	15.9 +/- 1.4	1.0	0.08
ENNB	1-200	0.998 +/- 0.001	11.4 +/- 3.7	1.0	0.01
ENNB1	1-200	0.997 +/- 0.001	15.4 +/-3.8	1.0	0.001
BEA	1-200	0.995 +/- 0.002	15.4 +/-4.3	1.0	0.001
FB2	10-200	0.993 +/- 0.003	13.9+/-4.7	10	0.17

Table S6. Validation results for linearity (linear range, correlation coefficient (r) and goodness-of-fit coefficient (g)), limit of quantification (LOQ), and limit of detection (LOD) of 24 mycotoxins in pig plasma.

Analyte	Linearity (*n* = 3 Different Days)			LOQ (ng/mL)	LOD (ng/mL)
	Linear Range (ng/mL)	r ± SD	g (%) ± SD		
ZEN	1-200	0.996 +/- 0.003	12.9 +/- 3.8	1.0	0.04
AZEL	1-200	0.995 +/-0.002	15.0 +/-1.2	1.0	0.08
AZAL	1-200	0.995+/-0.003	15.2 +/-3.6	1.0	0.05
BZAL	1-200	0.996 +/-0.001	10.7 +/-2.0	1.0	0.06
BZEL	1-200	0.996 +/-0.002	14.9 +/-3.7	1.0	0.11
ZAN	1-200	0.997 +/-0.001	16.3 +/-3.1	1.0	0.07
TEA	1-200	0.998 +/- 0.001	12.0 +/-4.9	1.0	0.12
AOH	1-200	0.997 +/-0.002	12.9 +/-5.2	1.0	0.05
AME	1-200	0.996+/-0.004	13.1+/-5.1	1.0	0.01
DON	1-200	0.998 +/- 0.002	13.9 +/- 4.4	1.0	0.21
DOM-1	1-200	0.997 +/-0.003	16.9 +/-2.5	1.0	0.35
3/15 ADON	1-200	0.998 +/- 0.001	9.3 +/-3.1	1.0	0.03
T2	1-200	0.998 +/-0.001	9.9 +/-1.6	1.0	0.05
HT2	1-100	0.993+/- 0.002	17.5 +/-2.8	1.0	0.54
T2G	2-200	0.995 +/- 0.003	14.4 +/-1.2	2.0	0.01
AFB1	1-200	0.996 +/-0.002	12.7 +/-2.8	1.0	0.01
AFM1	1-200	0.997 +/- 0.002	13.8 +/-5.6	1.0	0.001
OTA	1-200	0.993 +/-0.004	9.3+/-2.9	1.0	0.19
ENNA1	1-200	0.998 +/- 0.001	9.0 +/-0.9	1.0	0.005
ENNA	1-50	0.995+/-0.003	13.9 +/-3.2	1.0	0.18
ENNB	1-100	0.993+/-0.002	9.3 +/-1.2	1.0	0.01
ENNB1	1-100	0.998 +/- 0.001	15.9 +/-2.3	1.0	0.005
BEA	1-100	0.998 +/-0.000	16.6 +/-4.5	1.0	0.07

Note: SD = standard deviation; acceptance criteria: r ≥ 0.990 and g ≤ 20.

Table S7. Results of the within-day and between-day precision and accuracy experiments for 24 mycotoxins in pig plasma.

Analyte	Within-Day Precision and Accuracy (*n* = 6)						Between-Day Precision and Accuracy (*n* = 3 × 3)					
	Theoretical Concentration LOQ		Theoretical Concentration 10 ng/mL		Theoretical Concentration 100 ng/mL		Theoretical Concentration LOQ		Theoretical Concentration 10 ng/mL		Theoretical Concentration 100 ng/mL	
	Precision (RSD %)	Accuracy (%)	Precision (RSD %)	Accuracy (%)	Precision (RSD %)	Accuracy (%)	Precision (RSD%)	Accuracy (%)	Precision (RSD%)	Accuracy (%)	Precision (RSD %)	Accuracy (%)
ZEN	12.1	6.5	7.3	1	5.4	-1.5	13.4	2.7	7.5	2.4	5.7	-2.2
AZEL	6.5	19.7	13	2.3	7.4	6.6	35.4	-5.3	10.9	-0.7	11.6	1.5
AZAL	17.4	-14.5	7.7	-1.4	4.4	-0.4	20.9	-3.3	8.2	-5.2	8.1	-4.9
BZAL	5.2	7	3.5	8.2	2.9	8.9	9.3	6.2	3.9	6.6	10.7	4.2
BZEL	13.0	-9.2	5.1	-1.8	6.1	-2.4	15.9	-1.8	8.1	-4.9	6.6	-5.3
ZAN	11.7	-40.5	3.9	8.8	4.6	3.0	21.5	-32.4	3.6	9.8	7.1	0.4
TEA	2.8	19.0	3.2	8.2	3.6	4.5	10.7	18.2	3.4	8.7	5.8	4.8
AOH	17.6	-32.2	3.5	8.6	3.8	-0.4	26.9	-20.8	4.5	9.5	5.3	-3.1
AME	14.1	10.1	7.7	2.4	4.8	-12.3	18.7	-5.3	7.0	4.8	5.9	-11.9
DON	24.9	-8.0	6.9	1.0	5.6	-5.7	22.3	4.4	10.0	2.3	8	-4.6
DOM-1	17.5	-0.4	15.0	-3.3	5.9	-6.9	14.7	-2.0	14	-2.7	7.9	-7.3
3/15 ADON	15.6	7.5	5.2	5.2	6.4	-3.5	16.4	10.9	5.4	6.9	8.6	-3.5
T2	15.4	3.3	1.6	7.7	2.7	8.4	12.7	3.7	1.5	8.1	6.2	5.0
HT2	21.1	-29.8	6.5	-14.0	9.9	5.8	30.5	-21.4	10.0	-11.5	5.7	2.1
T2G	10.8	-3.8	7.8	6.9	7.0	1.3	23.8	-2.6	9.5	4.1	13.8	6.6
AFB1	13.1	-14.3	3.0	3.9	4.9	-2.0	16.4	-16.9	3.6	4.5	6.3	-2.8
AFM1	11.2	-38.8	10.6	-19.5	8.8	-15.6	28.0	-28.5	18.8	-7.1	20.2	-5.4
OTA	7.5	13.4	8.7	-13.3	4.5	-12.3	14.4	2.5	7.2	-12.5	9.2	-8.0
ENN A1	15.7	-11.3	12.6	-3.7	6.7	1.8	14.6	-2.3	10.8	-0.8	9.6	-0.9
ENNA	19.4	-1.0	9.4	-14.1	11.7	-14.7	41.7	-11.2	13.8	-6.8	13.5	-5.6
ENNB	16.7	-0.1	11.8	9.6	2.6	9.4	16.8	-1.4	13.9	-1.3	6.6	4.1
ENNB1	7.6	16.1	3.9	-0.1	3.5	-3.9	31.8	1.6	8.7	5.5	3.2	-3.2
BEA	13.3	-2.9	3.2	6.9	3.2	7.4	29.7	-6.3	2.3	7.2	11.9	8.5

Note: The acceptance criteria: Accuracy, ⩽ 1 ng/mL : −50% to +20%; 1 – 10 ng/mL: −30% to +10% ; ⩾ 10ng/mL: −20 to +10%. Within-day precision: RSD% < RSDmax with RSDmax for ⩾ 1 to < 10 ng/mL: < 25% and ⩾ 10 to < 100 ng/mL : < 15%. Between-day precision: the RSD% < RSDmax with RSDmax 22.6%, 32% and 45% for the respective concentrations of 100 ng/mL, 10 ng/mL and 1 ng/mL.

Table S8. Results of the within-day and between-day precision and accuracy experiments for 24 mycotoxins in pig faeces.

Analyte	Within-Day Precision and Accuracy (*n* = 6)						Between-Day Precision and Accuracy (*n* = 6)					
	Theoretical Concentration LOQ		Theoretical Concentration 10 ng/mL		Theoretical Concentration 100 ng/mL		Theoretical Concentration LOQ		Theoretical Concentration 10 ng/mL		Theoretical Concentration 100 ng/mL	
	Precision (RSD %)	Accuracy (%)	Precision (RSD %)	Accuracy (%)	Precision (RSD %)	Accuracy (%)	Precision (RSD%)	Accuracy (%)	Precision (RSD%)	Accuracy (%)	Precision (RSD %)	Accuracy (%)
ZEN	16.1	-21.2	3.7	-10.4	9.3	-3.2	21.2	-11.2	5.2	-10.4	9.7	-4.2
AZEL	6.9	1.6	7.2	-0.8	8.3	-1.7	28.0	4.0	6.1	1.3	6.8	-2.9
AZAL	3.3	10	6.5	2.5	1.3	-0.6	4.9	6.8	6.1	0.1	2.8	-2.4
BZAL	5.3	-17.6	7.9	-0.8	5.0	1.00	22.4	-10.1	12.1	-5.3	9.5	-2.0
BZEL	22.4	-29.0	2.8	-5.6	6.7	-4.7	37.9	-3.6	9.0	-0.5	5.3	-5.0
ZAN	21.6	-10.0	5.0	3.9	7.0	1.1	28.5	-19.5	9.0	0.6	7.8	2.9
TEA	11.0	18.2	6.7	-4.0	4.1	-7.6	27.7	12.5	8.5	-3.0	8.8	-3.6
AOH	14.1	-5.5	11.0	-3.4	5.9	-1.1	19.3	-5.9	11.9	-7.3	6.3	1.6
AME	19.4	-24.4	3.9	-9.2	8.4	4.6	39.2	-18.9	7.8	-8.5	8.3	4.0
DON	8.0	-8.7	5.6	-19.0	6.0	-18.9	7.7	-5.6	11.7	-12.4	12.7	-12.5
DOM-1	11.6	-4.0	9.7	-7.3	5.5	-2.8	10.5	-0.8	10.2	-7.0	7.3	-1.9
3/15 ADON	24.9	4.6	8.6	-13.6	7.9	-14.4	22.6	-0.8	7.5	-10.5	9.9	-8.6
T2	22.7	8.4	6.5	3.3	3.9	0.9	24.1	-0.5	6.2	2.8	4.1	3.1
HT2	7.7	1.1	7.0	1.0	7.2	-5.6	6.1	1.4	10.1	4.3	8.0	-2.0
T2G	22.2	-3.4	7.9	1.9	7.5	2.0	17.8	-0.1	8.6	-3.5	8.2	4.8
AFB1	18.6	-6.0	3.5	3.3	5.3	1.4	22.8	-9.9	5.6	1.6	4.1	1.8
AFM1	21.7	-1.6	2.2	-1.7	6.1	-4.0	29.9	6.0	1.9	-1.5	7.6	0.5
OTA	20.6	-8.8	4.4	-5.3	3.8	-3.8	18.8	-12.0	6.5	-4.1	5.3	-0.4
ENN A1	11.3	-10.7	6.6	9.9	7.0	6.5	25.3	0.5	11.4	3.2	10.9	-1.7
ENNA	21.5	-17.6	13.4	-5.8	5.7	-3.9	29.7	-22.8	15.7	0.8	13.0	1.5
ENNB	18.4	-7.2	6.6	6.0	4.3	2.3	21.7	4.5	7.3	5.4	15.4	-7.9
ENNB1	22.2	-9.1	5.7	-2.7	9.0	-8.6	30.8	-20.9	13.1	4.9	10.8	-6.1
BEA	20.8	-0.8	13.3	-7.9	8.9	1.0	25.6	15.8	17.0	1.4	12.4	-0.7

Table S9. Results of the within-day and between-day precision and accuracy experiments for 24 mycotoxins in pig urine.

Analyte	Within-Day Precision and Accuracy (*n* = 6)						Between-Day Precision and Accuracy (*n* = 6)					
	Theoretical Concentration LOQ		Theoretical Concentration 10 ng/mL		Theoretical Concentration 100 ng/mL		Theoretical Concentration LOQ		Theoretical Concentration 10 ng/mL		Theoretical Concentration 100 ng/mL	
	Precision (RSD %)	Accuracy (%)	Precision (RSD %)	Accuracy (%)	Precision (RSD %)	Accuracy (%)	Precision (RSD%)	Accuracy (%)	Precision (RSD%)	Accuracy (%)	Precision (RSD %)	Accuracy (%)
ZEN	12.0	-5.5	8.8	-5.8	2.4	-4.3	10.1	-6.2	7.2	-5.4	2.8	-3.1
AZEL	10.7	-12.6	11.6	-10.5	4.6	-7.4	12.6	-5.6	10.0	-8.1	6.7	-4.2
AZAL	9.8	14.3	12.2	-10.6	4.1	-15.1	7.9	12.1	9.7	-9.5	9.5	-8.8
BZAL	6.5	15.4	4.3	3.1	3.2	-1.3	6.5	15.4	4.3	3.1	3.2	-1.3
BZEL	13.5	-18.3	1.8	5.5	4.2	-7.5	12.1	-21.4	1.7	6.1	4.6	-5.8
ZAN	5.6	-4.0	2.0	7.9	6.4	-4.0	16.1	-8.5	3.4	7.1	5.2	-3.2
TEA	21.6	-17.1	6.7	-10.5	7.5	-0.2	18.9	-10.9	8.2	-7.5	7.1	1.0
AOH	24.8	-7.0	14.0	-8.5	7.2	2.6	32.1	-15.0	11.6	-7.9	10.8	-3.1
AME	21.3	-1.5	6.3	1.6	6.5	2.7	20.3	-5.0	17.0	-7.1	14.8	-4.8
DON	10.7	-14.3	4.8	-2.7	5.9	1.4	10.7	-14.3	4.8	-2.7	5.9	1.4
DOM-1	12.6	1.3	13.6	3.4	9.6	-2.9	9.0	2.1	12.0	-0.5	9.3	2.5
3/15 ADON	17.4	14.5	4.9	3.5	5.5	-0.3	14.8	11.5	5.0	2.6	7.2	-1.7
T2	4.7	17.2	5.4	2.0	2.8	5.7	11.4	10.2	5.3	0	4.3	3.2
HT2	20.9	-10.0	8.6	-16.9	3.4	-7.4	23.1	-11.1	10.2	-17.4	3.4	-7.8
T2G	24.4	-8.4	5.1	4.7	4.8	-1.7	28.2	-14.1	12.6	-2.3	8.3	-5.0
AFB1	6.0	-8.0	3.0	2.0	1.7	-1.2	16.0	-12.8	3.4	1.5	1.9	-0.7
AFM1	7.0	19.2	7.6	4.9	5.6	-9.7	22.1	5.2	6.8	2.6	8.8	-8.4
OTA	10.7	-1.0	5.6	-2.8	4.7	2.9	9.5	2.1	4.4	-3.3	4.3	2.4
ENN A1	1.8	10.3	4.0	1.9	6.3	-5.2	5.6	6.8	3.3	1.7	5.5	-6.8
ENNA	7.5	-6.1	4.0	-15.1	9.4	-11.7	6.4	-5.1	4.9	-12.7	8.7	-11.1
ENNB	3.4	9.1	4.4	-0.4	8.2	-11.9	10.1	2.3	3.7	-1.2	6.4	-10.6
ENNB1	3.5	14.8	2.8	2.7	7.1	-1.2	6.0	12.6	3.1	2.4	5.8	-1.1
BEA	1.7	-1.7	7.5	-5.7	9.3	-8.2	6.8	-6.4	6.2	-4.7	11.1	-7.9

Table S10. Results of the within-day and between-day precision and accuracy experiments for 24 mycotoxins in broiler chicken plasma.

Analyte	Within-Day Precision and Accuracy (*n* = 6)						Between-Day Precision and Accuracy (*n* = 6)					
	Theoretical Concentration LOQ		Theoretical Concentration 10 ng/mL		Theoretical Concentration 100 ng/mL		Theoretical Concentration LOQ		Theoretical Concentration 10 ng/mL		Theoretical Concentration 100 ng/mL	
	Precision (RSD %)	Accuracy (%)	Precision (RSD %)	Accuracy (%)	Precision (RSD %)	Accuracy (%)	Precision (RSD%)	Accuracy (%)	Precision (RSD%)	Accuracy (%)	Precision (RSD %)	Accuracy (%)
ZEN	16.8	17.1	4.7	-6.5	4.0	-3.7	22.3	8.0	5.4	-5.2	4.9	-3.3
AZEL	10.2	-16.3	2.9	-15.7	9.5	-8.7	29.6	-6.9	10.6	-11.8	8.9	-4.1
AZAL	17.4	-34.3	5.5	-19.7	7.8	-9.4	36.0	-25.4	11.9	-13.3	8.0	-5.4
BZAL	14.6	-9.0	10.3	-19.5	8.6	-16.5	18.0	-0.2	13.8	-14.1	11.4	-10.2
BZEL	21.1	7.8	11.1	-15.0	8.8	-16.5	22.9	2.1	11.8	-9.9	13.6	-8.8
ZAN	21.2	-2.1	8.2	-8.1	8.1	-2.3	21.1	-8.2	6.5	-6.4	7.7	-3.1
TEA	22.3	-7.5	11.5	-19.7	8.0	-3.6	17.8	0.4	13.6	-13.3	11.4	-5.3
AOH	21.1	-3.3	9.6	-0.2	8.6	2.7	18.2	0.2	12.1	-5.4	8.7	0.5
AME	9.2	-9.3	4.5	8.7	8.9	6.8	19.7	-8.2	5.2	5.6	15.6	8.6
DON	22.7	14.0	9.4	1.8	4.5	-4.0	18.6	8.9	9.0	-0.6	4.7	-4.5
DOM-1	12.8	-9.4	6.9	2.9	5.4	-9.7	15.7	-14.5	8.1	2.7	6.8	-5.7
3/15 ADON	3.9	19.6	13.2	2.7	6.4	-19.1	12.6	14.7	13.8	-2.9	13.2	-10.7
T2	3.5	2.2	7.7	-5.9	5.3	-2.0	6.7	-1.4	7.8	-3.9	7.9	-3.0
HT2	16.9	-6.0	4.0	-19.6	6.0	-18.8	19.4	-11.4	10.8	-14.5	12.1	-11.2
T2G	4.5	5.6	10.6	7.7	7.4	0.0	4.5	5.6	8.3	9.1	7.1	3.6
AFB1	4.0	7.0	3.8	-3.2	3.3	-3.2	10.1	0	4.4	-3.9	4.7	-3.8
AFM1	17.4	-28.4	6.1	-17.1	8.3	-19.9	17.4	-28.4	10.2	-13.4	12.0	-12.5
OTA	24.2	-7.2	3.8	-2.7	2.6	-5.0	23.8	-15.1	4.4	-4.8	3.1	-5.7
ENN A1	10.2	-30.7	12.7	0.8	8.9	-15.4	24.2	-17.3	10.6	-1.7	13.5	-7.5
ENNA	20.4	-15.1	9.7	2.0	8.0	-12.3	14.2	-14.1	8.1	1.2	9.8	-8.1
ENNB	2.1	5.6	3.5	-8.0	4.4	-2.6	7.7	2.1	3.2	-7.6	6.2	1.0
ENNB1	13.1	13.1	8.0	-2.9	8.9	0.5	24.6	-2.8	7.6	0.7	10.0	-1.9
BEA	3.8	3.3	10.9	-15.9	9.0	-11.1	16.0	-2.1	12.9	-8.9	12.6	-6.6

Table S11. Results of the within-day and between-day precision and accuracy experiments for 24 mycotoxins in broiler chicken excreta.

Analyte	Within-Day Precision and Accuracy (*n* = 6)						Between-Day Precision and Accuracy (*n* = 6)					
	Theoretical Concentration LOQ		Theoretical Concentration 10 ng/mL		Theoretical Concentration 100 ng/mL		Theoretical Concentration LOQ		Theoretical Concentration 10 ng/mL		Theoretical Concentration 100 ng/mL	
	Precision (RSD %)	Accuracy (%)	Precision (RSD %)	Accuracy (%)	Precision (RSD %)	Accuracy (%)	Precision (RSD%)	Accuracy (%)	Precision (RSD%)	Accuracy (%)	Precision (RSD %)	Accuracy (%)
ZEN	20.0	-23.3	6.1	8.5	2.8	7.3	15.7	-23.6	8.6	2.6	10.4	3.0
AZEL	11.1	-16.6	4.4	7.3	3.8	7.8	12.6	-17.1	15.5	-3.7	12.7	-0.2
AZAL	14.3	7.3	4.5	0.7	4.9	4.3	19.3	-1.1	8.7	-5.0	12.3	-2.7
BZAL	8.6	13.5	3.3	2.8	3.4	6.3	21.7	2.2	11.7	-4.3	13.3	-2.3
BZEL	16.6	-3.4	3.7	2.4	2.5	3.5	33.7	1.0	7.5	-0.5	6.2	-0.5
ZAN	13.1	-7.1	2.0	-0.1	2.5	6.5	19.0	-0.9	2.2	1.1	8.6	2.5
TEA	20.1	-15.9	8.4	-13.5	1.8	8.6	20.8	-16.8	9.5	-8.0	9.1	2.7
AOH	13.1	-20.4	13.7	3.1	3.2	8.2	16.9	-16.0	16.5	-4.0	14.4	0.5
AME	10.3	6.2	6.4	1.9	7.0	-14.6	16.6	-0.1	8.7	0	12.6	-10.2
DON	18.5	16.2	6.4	7.3	4.4	2.7	20.1	18.4	8.8	3.8	6.1	3.4
DOM-1	10.5	-2.2	3.3	7.2	2.0	8.0	27.4	-17.2	6.0	4.0	9.1	4.2
3/15 ADON	9.3	7.0	12.5	-3.2	10.4	-14.3	15.4	-0.1	11.8	-1.8	14.9	-14.6
T2	6.9	-10.5	2.3	0.6	1.9	4.4	13.0	-10.8	4.1	-0.6	3.4	4.5
HT2	15.3	-13.6	7.0	-9.8	3.6	0.7	15.3	-13.6	15.0	-4.7	7.0	0.7
T2G	17.0	7.7	7.2	-1.7	7.0	7.7	13.3	8.7	6.4	-0.4	7.0	5.4
AFB1	10.7	-25	1.6	-1.2	2.4	1.5	13.4	-23.2	3.4	0.6	2.9	1.6
AFM1	21.0	-15.3	1.7	4.9	4.4	-10.6	14.2	-1.2	14.5	-7.7	19.0	-4.5
OTA	12.1	-4.4	6.3	-2.5	5.1	-2.2	44.7	-25.0	7.0	-0.8	8.9	-0.2
ENN A1	11.9	-30.8	2.6	4.8	1.0	5.5	23.9	-20.8	2.5	5.3	11.1	-0.4
ENNA	21.9	12.5	7.7	-17.4	6.2	-17.1	23.9	0.9	13.5	-10.5	9.4	-12.5
ENNB	20.0	-16.1	2.2	4.9	1.1	3.7	20.0	-14.2	2.4	5.5	8.8	0.6
ENNB1	8.0	-14.5	3.6	5.3	3.2	4.0	23.1	-20.9	4.7	4.4	13.4	-5.2
BEA	21.4	-8.5	1.9	8.2	2.1	8.8	43.7	-17.5	6.2	7.4	10.9	1.0
FB2	-	-	5.6	3.9	8.5	-0.4	-	-	4.7	3.6	7.9	0.4

Table S12. Mycotoxins, phase I and II and other metabolites and accurate mass used for UHPLC-HRMS analysis.

Mycotoxins and Phase I Metabolites	Accurate Mass (Da)	Phase II Metabolites	Accurate Mass (Da)
**Deoxynivalenol**	296.1260	**Deoxynivalenol-glucuronide (DON-GlcA)**	472.1581
**De-epoxy-deoxynivalenol**	280.1311	**De-epoxy-deoxynivalenol glucuronide**	456.1632
**3/15-acetyldeoxynivalenol**	338.1366	**Deoxynivalenol-sulphate (DON-S)**	376.0828
**Aflatoxin B1**	312.0634	**Deoxynivalenol-di-sulphate**	456.0396
**Aflatoxicol (AFL)**	314.0790	**3/15-acetyl-deoxynivalenol-sulphate**	418.0934
**Aflatoxin M1**	328.0538	**Zearalenone-glucuronide (ZEN-GlcA)**	494.1788
**AFP1**	298.0477	**Zearalenone-di-glucuronide**	670.2109
**AFQ1**	328.0583	**α/β-zearalenol- or zearalanone-glucuronide**	496.1945
**AFB2a**	330.0740	**Zearalenone-sulphate**	398.1035
**AFB1-dihydrodiol**	346.2910	**α/β-zearalenol- or zearalanone-sulphate**	400.1192
**Ochratoxin A**	403.0823	**α/β-zearalenol-sulphate**	402.1348
**OTα**	256.0139		
**OTB**	369.1212		
**4/5/7/9-OH-OTA**	419.0772		
**zearalenone**	318.1467		
**zearalanone**	320.1624		
**α-zearalenol**	320.1624		
**α-zearalanol**	322.1780		
**β-zearalenol**	320.1624		
**β-zearalanol**	322.1780		
**[$^{13}C_{15}$]-Deoxynivalenol**	311.1763		
**[$^{13}C_{17}$]-Aflatoxin B1**	329.1204		
**[$^{13}C_{20}$]-Ochratoxin A**	423.1494		
**[$^{13}C_{18}$]-Zearalenone**	336.2071		

Table S13. Relationship between European limits in feed and the dose administered.

Mycotoxin	European Limit (mg/kg Feed)	Dose of Mycotoxin Per kg BW (mg/kg BW) / Daily Feed Consumption Per kg BW (kg/kg BW)
OTA	0.1	0.25/0.100 ➔ 2.5 mg/kg feed
DON pig	0.9	0.036/0.040 ➔ 0.9 mg/kg feed
DON broiler chicken	5	0.5/0.100 ➔ 5 mg/kg feed
AFB1	0.02	2/0.100 ➔ 20 mg/kg feed
ZEN piglets and gilts	0.1	3/0.040 ➔ 75 mg/kg feed
